# Higher Throughput Assays for Understanding the Pathogenicity of Variants of Unknown Significance in the *RPE65* Gene

**DOI:** 10.1167/iovs.66.13.10

**Published:** 2025-10-06

**Authors:** Leila Azizzadeh Pormehr, Kannan Vrindavan Manian, Ha Eun Cho, T. Michael Redmond, Jason Comander

**Affiliations:** 1 Ocular Genomics Institute, Berman-Gund Laboratory for the Study of Retinal Degenerations, Massachusetts Eye and Ear, Harvard Medical School, Boston, Massachusetts, United States; 2 Laboratory of Retinal Cell & Molecular Biology, National Eye Institute, National Institutes of Health, Bethesda, Maryland, United States

**Keywords:** *RPE65*, variant of unknown significance, inherited retinal diseases, retinitis pigmentosa, voretigene neparvovec

## Abstract

**Purpose:**

RPE65 is a key enzyme in the visual cycle that regenerates 11-*cis*-retinal. Mutations in *RPE65* cause a retinal dystrophy that is treatable with an approved gene therapy. Variants of unknown significance (VUS) on genetic testing can prevent patients from obtaining a firm genetic diagnosis and accessing gene therapy. Because most *RPE65* mutations have a low protein expression level, this study developed and validated multiple methods for assessing the expression level of *RPE65* variants. This functional evidence is expected to aid in reclassifying *RPE65* VUS as pathogenic, which can improve the diagnosis and treatment of *RPE65* patients.

**Methods:**

Thirty different variants of *RPE65* (12 pathogenic, 13 VUS, five benign) were cloned into lentiviral expression vectors. Protein expression levels were measured after transient transfection or in stable cell lines, using western blots and immunostaining with flow cytometry. Then, a pooled, high-throughput, fluorescence-activated cell sorting (FACS) assay with a next-generation sequencing–based readout was used to assay pools of *RPE65* variants.

**Results:**

A high correlation was observed between protein levels measured by western blot, flow cytometry, and the pooled FACS assay. Using these assays, we confirmed and extended *RPE65* variant data, including that Pro111Ser has a low, pathogenic expression level. There was a high correlation between *RPE65* expression and previously reported enzyme activity levels; further development of a high-throughput enzymatic activity assay would complement these expression data.

**Conclusions:**

This scalable approach can be used to solve patient pedigrees with VUS in *RPE65*, facilitating treatment and providing *RPE65* structure–function information.

One of the primary goals of human genetics is to understand how genetic variation affects the function of genes and contributes to disease development.^1^ In fact, of more than 4 million missense identified variants, only about 2% have been definitively classified as pathogenic or benign. Most missense variants are classified as variants of unknown significance (VUS).^2^ The current study was motivated by the observation that genetic testing for inherited retinal diseases (IRDs) gives ambiguous or negative results about one-third of the time, often due to VUS.^3^^–^^7^ Proper pathogenicity classification of new or rare variants is important for a conclusive molecular diagnosis and the medical management of patients.^8^ This problem can have clinical consequences, preventing familial risk assessment, family planning, and access to an approved gene therapy or other investigational gene-specific therapies. In IRDs, this problem of VUS is particularly impactful for the *RPE65* gene, which was the first gene to have a corresponding U.S. Food and Drug Administration (FDA)-approved gene therapy. Indeed, genetic testing results containing a *RPE65* VUS can prevent patients from obtaining treatment with an FDA-approved gene therapy, voretigene neparvovec (Luxturna; Spark Therapeutics, Philadelphia, PA, USA).^9^^,^^10^ Mutations in the *RPE65* gene account for 0.6% to 6% of retinitis pigmentosa and 3% to 16% of Leber congenital amaurosis (LCA)/early onset retinal dystrophy (eoRD) cases,^11^ and patients are eligible for treatment only if they have documented biallelic pathogenic or likely pathogenic mutations.^12^

Although a number of computational tools can be used to predict pathogenicity,^2^^,^^13^^,^^14^ they are not highly accurate.^15^^,^^16^ Even with advances in computational algorithms,^17^^–^^19^ the level of accuracy may not be high enough when making medical decisions, including the decision to expose a patient to a gene therapy surgery specific to a certain genetic cause of disease. Additionally, American College of Medical Genetics and Genomics (ACMG) guidelines do not allow a definitive diagnosis based on computational predictions alone.^20^ As a result, the use of validated, laboratory-based functional assays is considered strong evidence toward the reclassification of VUS into pathogenic variants.^2^^,^^6^^,^^7^^,^^13^^–^^20^

Although the traditional method of investigating variant pathogenicity is to test one variant at a time, the benefits of producing this information at scale have resulted in the field of “functional genomics,” in which parallelized, higher throughput assays are used to assay pools of variants. Widespread use of functional genomics could improve the accuracy of variant interpretation in genes with both known and unknown associations with disease, generate information and reagents needed to test therapeutic agents, and inform the development of analytical tools for predicting variant pathogenicity. Therefore, the purpose of this study was to develop and validate higher throughput expression assays for *RPE65* to provide expression information on a panel of *RPE65* variants at a higher accuracy than can be achieved by bioinformatic estimates alone.

Mechanistically, *RPE65* plays the central role in the retinoid cycle^21^^–^^23^ and encodes RPE65 retinol isomerase. The retinoid cycle enzymatic pathway allows continuous vision by regeneration of 11-*cis*-retinal from all-*trans*-retinal, which becomes part of the main chromophore of phototransduction in photoreceptor cells.^24^^–^^26^ More than 230 missense mutations lacking a clear pathogenicity classification or classified as VUS have been reported in public databases for *RPE65* (ClinVar, https://www.ncbi.nlm.nih.gov/clinvar/?term=RPE65). Many of these variants introduce missense mutations that affect the protein expression level or enzymatic activity, including changes in protein localization or stability.^27^^–^^29^ For example, missense mutation of G40S caused a reduction in catalytic activity to 2% of wild-type (WT) levels and reduced protein levels to less than 40% of WT levels.^23^^,^^30^^,^^31^

A traditional method for quantifying protein levels in cells is the western blot, which is semiquantitative when calibrated, but it is low throughput and has a limited dynamic range. More scalable quantification techniques can include the use of fluorescent protein fusions (variant abundance by massively parallel sequencing [VAMP-seq]),^32^ flow cytometry,^33^ split luciferase systems,^34^ and barcoded transcriptional reporters.^35^ Flow cytometry is particularly suited to higher throughput assays implemented as pooled assays, as individual cells from a library can be rapidly separated for analysis. Fluorescence from immunostaining or from engineered fluorescent proteins can be used to sort cells based on the expression of cell surface and/or intracellular proteins. By combining fluorescence-activated cell sorting (FACS) and next-generation sequencing (NGS), it is possible to obtain a more comprehensive understanding of thousands of variants.^36^ Although many functional genomics studies have used tags to facilitate protein detection,^37^^,^^38^ this study used only the native to human RPE65 protein sequence to reduce the uncertainty that genetically encoded tags could disrupt the fidelity of the assays in a manner that is difficult to detect. We hypothesized that cross-validating different analytical techniques with different antibodies and across a range of *RPE65* variants would evaluate the specificity and reproducibility of the reagents and allow for the development of a higher throughput RPE65 protein expression assay based on flow cytometry.

## Materials and Methods

### Selection of *RPE65* Variants for Study

Thirty different *RPE65* variants were selected spanning a variety of pathogenicity levels: 12 pathogenic, 13 VUS, 5 benign (see Supplementary Table S1 for the Human Genome Variation Society [HGVS] nomenclature). WT *RPE65* was also included. Among the variants that underwent analysis by multiple assays (Table 1), the negative control mutants that are known to have low expression levels included G40S, R515W, G104V, P25L, P363T, G244V, and R124*. Y368H has conflicting expression level data and is known to have low activity. H241L and Y239D were selected as known mutations in the active site region. C112Y was reported as homozygous in IRD pedigrees,^39^^,^^40^ which this study considered to be likely pathogenic. The positive control variants were A434V and N321K, which are known to have normal^30^ or slightly decreased^23^ activity and have a high maximum allele frequency in humans: A434V, 7.7% in African/African Americans; N321K, 3.5% in South Asians (gnomAD database v4.1.0). K294T was a likely benign variant, with somewhat conflicting activity levels reported.^23^^,^^30^ The variants N248S, V189I, T94A, and T390I were selected from a panel of VUS that were detected in IRD patients (provided by Spark Therapeutics).

**Table 1.** Comparison of the Expression Levels of Different Known *RPE65* Variants Between the Results in This Study and Those in Previous Studies

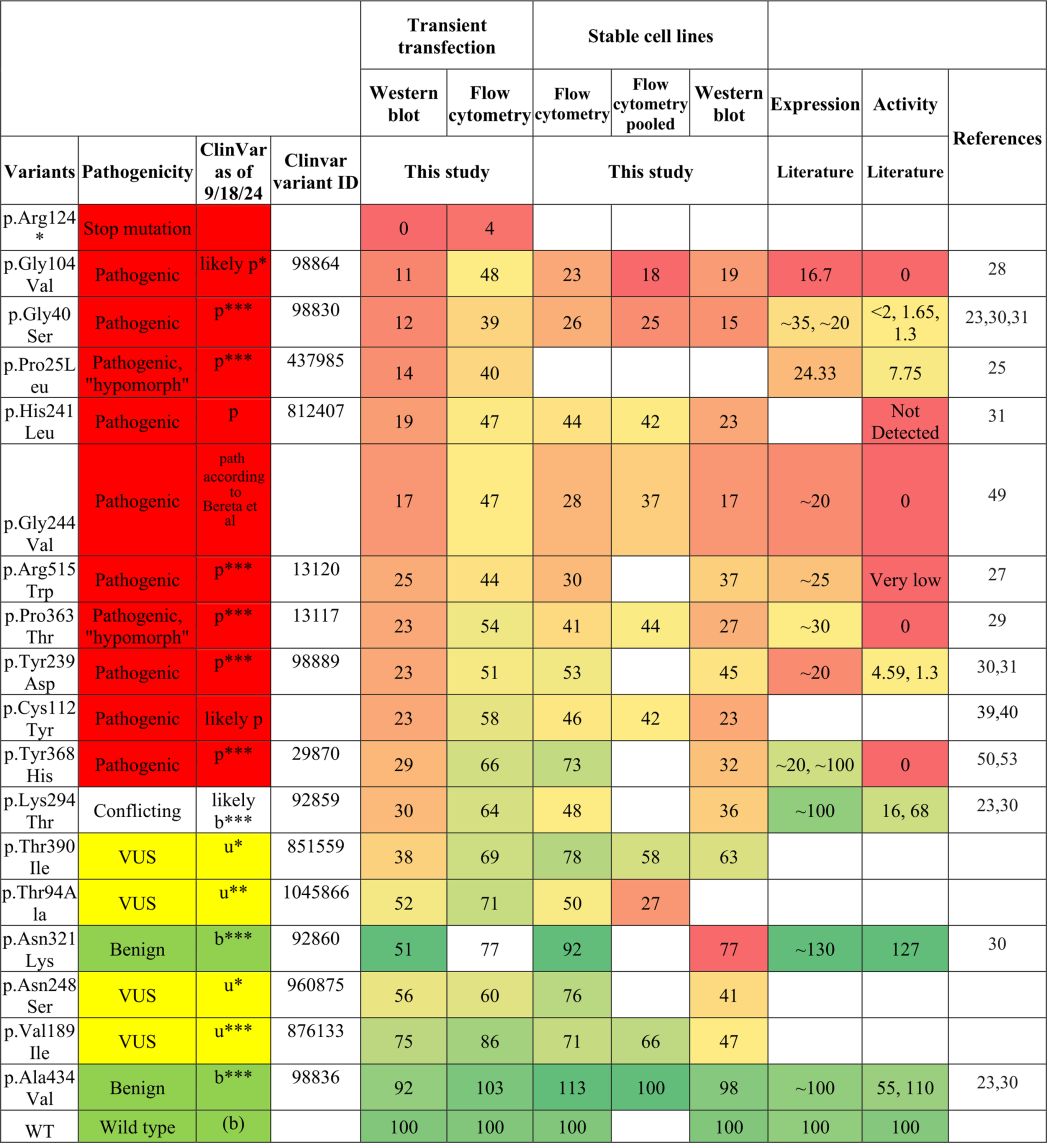	

Results are expressed as a percent of wild type. The color code for values is *green* = 100 and *red* = 0. The color code for pathogenicity is *green* = benign (b), *yellow* = VUS (u), and *red* = pathogenic (p). Asterisks denote the number of stars in the ClinVar evidence rating.

A second batch of variants was selected for testing by flow cytometry analysis only (Table 2). A434=, T385=, and E352= are synonymous variants hypothesized to behave as wild type. T86N, S533T, T105N, P111S, and N135K were selected because, at the time of initiation of this project, the variants were identified as VUS by the *RPE65* Variant Curation Expert Panel (Lori Sullivan, personal communication). G193S was selected as a VUS from the ClinVar database. L450V is a potential hypomorphic allele from our institution's patient cohort. D477G is a known dominant pathogenic mutation that produces a distinct phenotype compared to recessive *RPE65* mutations.^23^ H527R is a known mutation in the active site region.

**Table 2.** Expression Levels of Additional *RPE65* Variants Assayed Using the Transient Transfection Method

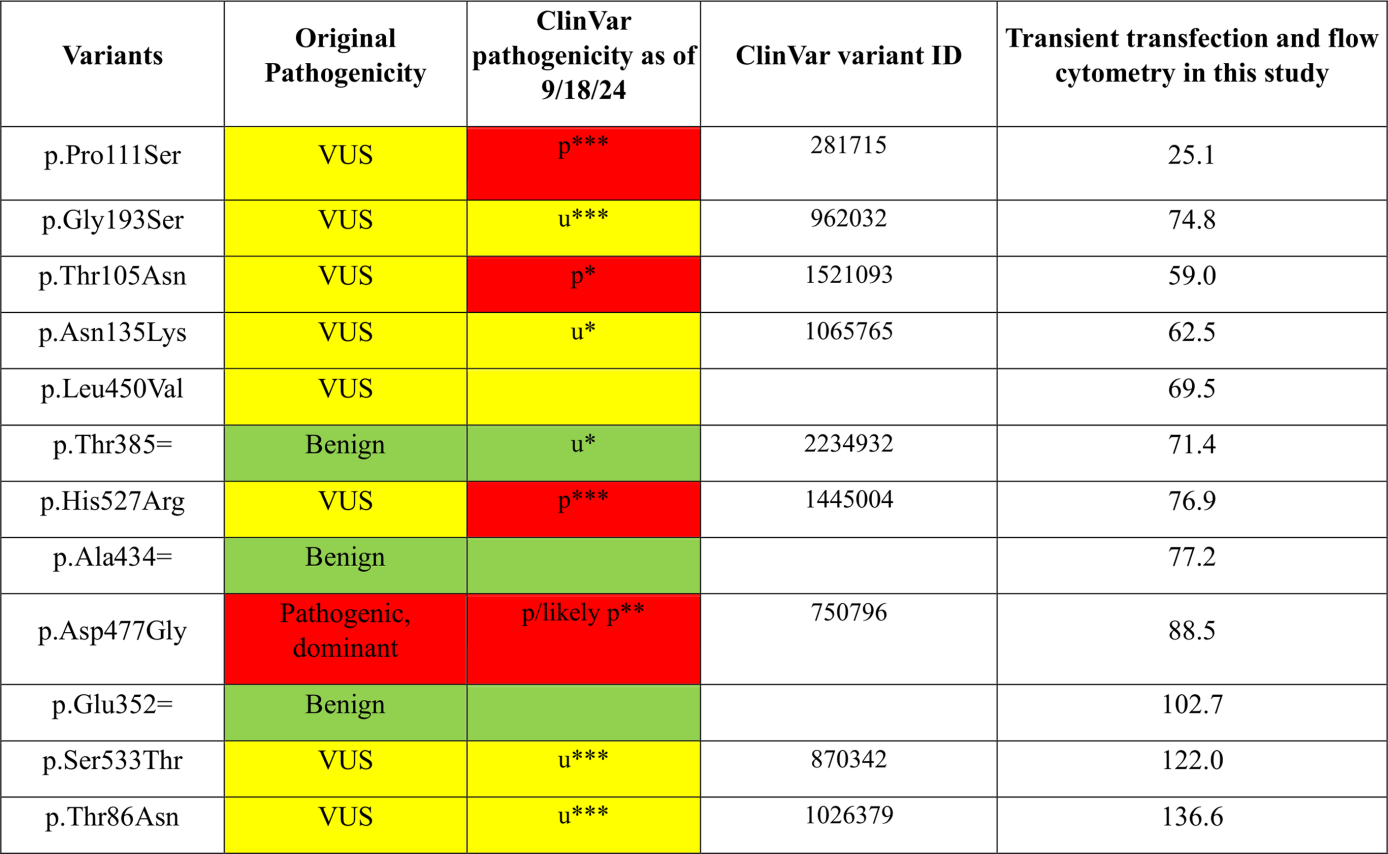	

The color code for pathogenicity is *green* = benign (b), *yellow* = VUS (u), *red* = pathogenic (p). Asterisks denote the number of stars in the ClinVar evidence rating.

### Plasmid Construction

The human *RPE65* cDNA (NM_000329.3, without untranslated regions) was amplified and cloned into the NheI and BamH1 site of pMT_025 lentiviral expression vector (158579; Addgene, Watertown, MA, USA).^41^ Mutagenesis was used to create the DNA changes as listed in Supplementary Table S1. NGS or Sanger sequencing was performed to verify the correct sequence in all plasmids.

### Transient Transfection of Variants

Thirty different variants of *RPE65* were used for transfection in HEK293T cells (CRL-3216; American Type Culture Collection, Manassas, VA, USA). For transfection, HEK293T cells were seeded in six-well (C6) or 12-well (C12) plates at a density of 4.3 × 10^4^ cells/cm^2^. The cells were maintained in Dulbecco’s Modified Eagle Medium (1-995-065; Thermo Fisher Scientific, Waltham, MA, USA) supplemented with 10% fetal bovine serum (SH3007103; Thermo Fisher Scientific). At 70% to 80% confluency, 2.5 µg plasmids were transfected per C6 well using Lipofectamine 3000 Transfection Reagent (L3000008; Thermo Fisher Scientific) according to the manufacturer's instructions. Cells were assayed 48 hours post-transfection.

### Generation of Stable Cell Lines for *RPE65* Variants

Lentiviruses were prepared by transfecting HEK293FT cells with psPAX2 (12260; Addgene), pMD2.G (12259; Addgene), and pMT_025 *RPE65* variant using Lipofectamine LTX Reagent (Thermo Fisher Scientific) according to the manufacturer's instructions. The viral supernatant was harvested, concentrated, and transduced into HEK293T cells at a multiplicity of infection (MOI) of <0.3. The transduced cells were selected using puromycin to generate stable lines (see also Supplementary Methods).

### Western Blotting

Whole-cell lysates were prepared using radioimmunoprecipitation assay (RIPA) buffer from the transient and stable cell lines. After quantification, the lysates underwent sodium dodecyl sulfate–polyacrylamide gel electrophoresis (SDS-PAGE; 4%–20% Invitrogen Tris-Glycine Gel; Thermo Fisher Scientific), followed by dry transfer using an Invitrogen iBlot 2 system. Blots were incubated with primary antibodies for RPE65 and β-actin overnight at 4°C followed by respective secondary antibodies (see Supplementary Methods for antibody details). Band intensities were detected using the Odyssey CLx Imager (LICORbio, Lincoln, NE, USA) (also see Supplementary Methods).

### Quantification of *RPE65* Expression Levels by Flow Cytometry

Initial experiments used the PETLET RPE65 antibody (not shown; from the T. Michael Redmond laboratory at the National Eye Institute), but the following experiments were conducted with commercially available antibodies that are more broadly available. At 48 hours, cells were trypsinized, washed, fixed in 4% paraformaldehyde (PFA), washed, permeabilized with 0.02% saponin, and blocked in 3% BSA in PBS. Cells were then incubated with primary antibodies described above at a final concentration of 1:1500 for 1 hour. After washing, Alexa Fluor 488 secondary antibodies were used for detection on a MACSQuant Flow Cytometer (Miltenyi Biotec, Bergisch Gladbach, Germany) (also see Supplementary Methods).

### Pooled Quantification of *RPE65* Expression Levels by FACS and NGS

Stable cell lines expressing *RPE65* variants (*n* = 16 variants) were pooled together, fixed using 4% PFA, and stained for FACS as described previously for flow cytometry. The stained cells were sorted on a Sony SH800 Cell Sorter or MA900 Multi-Application Cell Sorter (Sony Biotechnology, San Jose, CA, USA) by their *RPE65* expression level, where the “high” and “low” gates contained the top and bottom 18% of fluorescent intensity, respectively. The cells were lysed, and the genomic DNA from *RPE65*^high^, *RPE65*^low^, and unsorted cells was un-crosslinked (manuscript in preparation) and extracted. The open reading frame of the lentiviral transgenes was amplified and quantified using NGS and compared to flow cytometry expression results. Briefly, the open reading frame containing the *RPE65* variants was amplified using primers XY304 (ATTCTCCTTGGAATTTGCCCTTT) and XY305 (CATAGCGTAAAAGGAGCAACA). NGS libraries (170–280 bp) were created using fragmentation and sequenced on a MiSeq Sequencing System (Illumina, San Diego, CA, USA). Fastq files were aligned to a reference sequence containing the pMT025–*RPE65* plasmid. Variants were quantified using bam-readcount^42^ and normalized to read depth. The relative expression level for each variant in the pool was calculated as the variant count in the high gate divided by the count in the low gate.^33^

### Effect of L450V Mutation on RPE65 Isomerization Activity

FreeStyle 293-F Cells (R79007; Thermo Fisher Scientific) were cultured and transfected according to the published protocol.^23^ Briefly, the cells were transfected with 30 µg of pVitro2^23^ bicistronic plasmid (containing open reading frames [ORFs] for dog *RPE65* and bovine *RLBP1*) and 30 µg of pVitro3^23^ bicistronic plasmid (containing ORFs for bovine *LRAT* and bovine *RDH5*) using 293fectin Transfection Reagent (12347019; Thermo Fisher Scientific). All-trans retinol was added 24 h post-transfection and after 7 h the cells were harvested. The cells were harvested by centrifugation and processed for retinoid extraction, followed by high-performance liquid chromatography analysis.

## Results

### Measuring the Linearity of Western Blot Detection of RPE65 and β-Actin

**Figure 1.**
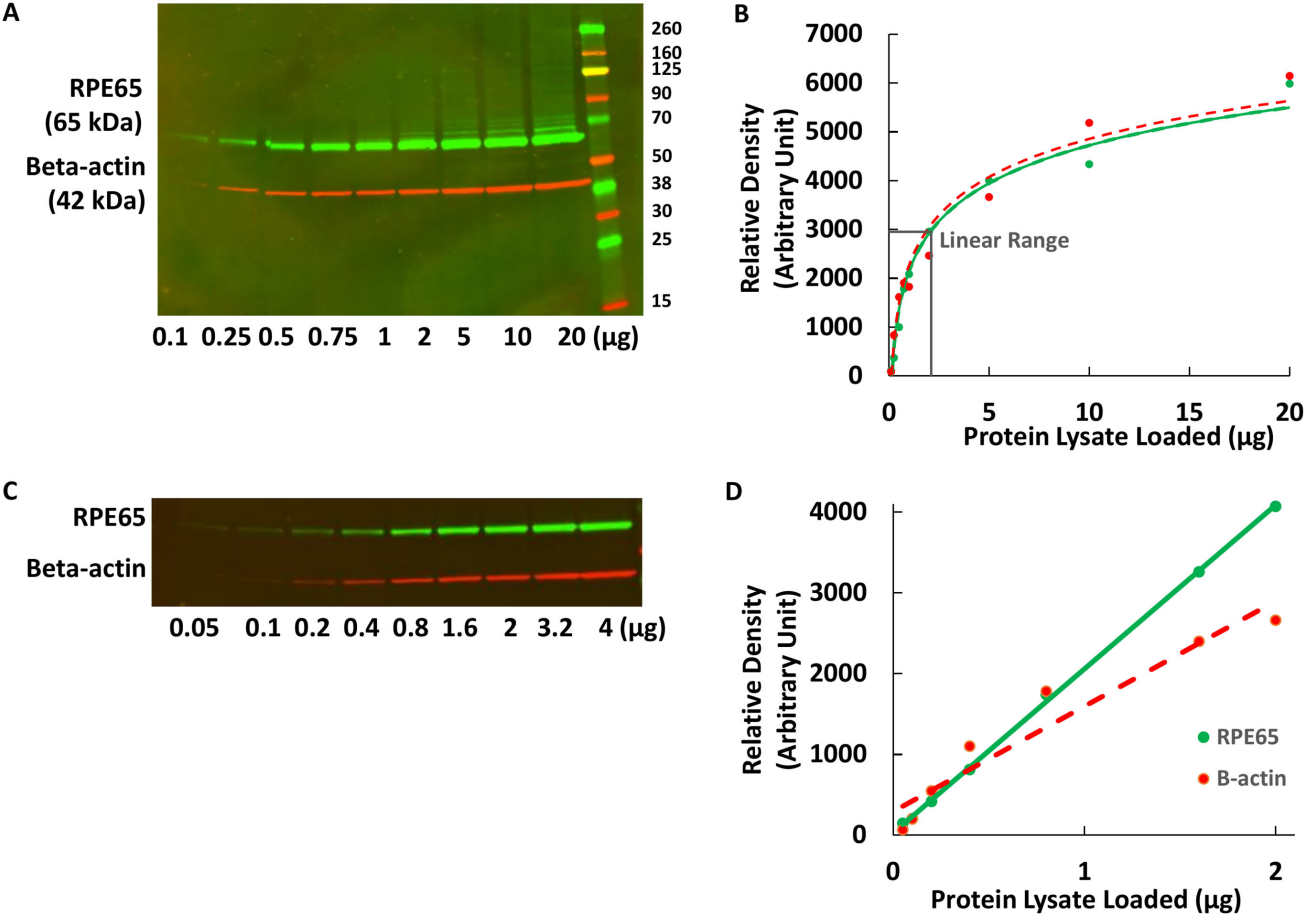
(**A**–**D**) Linear range determination for RPE65 and β-actin by western blotting: 0.1 to 20 µg lysate (**A**) and 0.05 to 4 µg lysate (**C**), with corresponding densitometry results shown (**B**, **D**). For **D**, the intensity was linearly correlated with the protein loading amount for RPE65 (*r* = 0.99) and for β-actin (*r* = 0.96).

**Figure 2.**
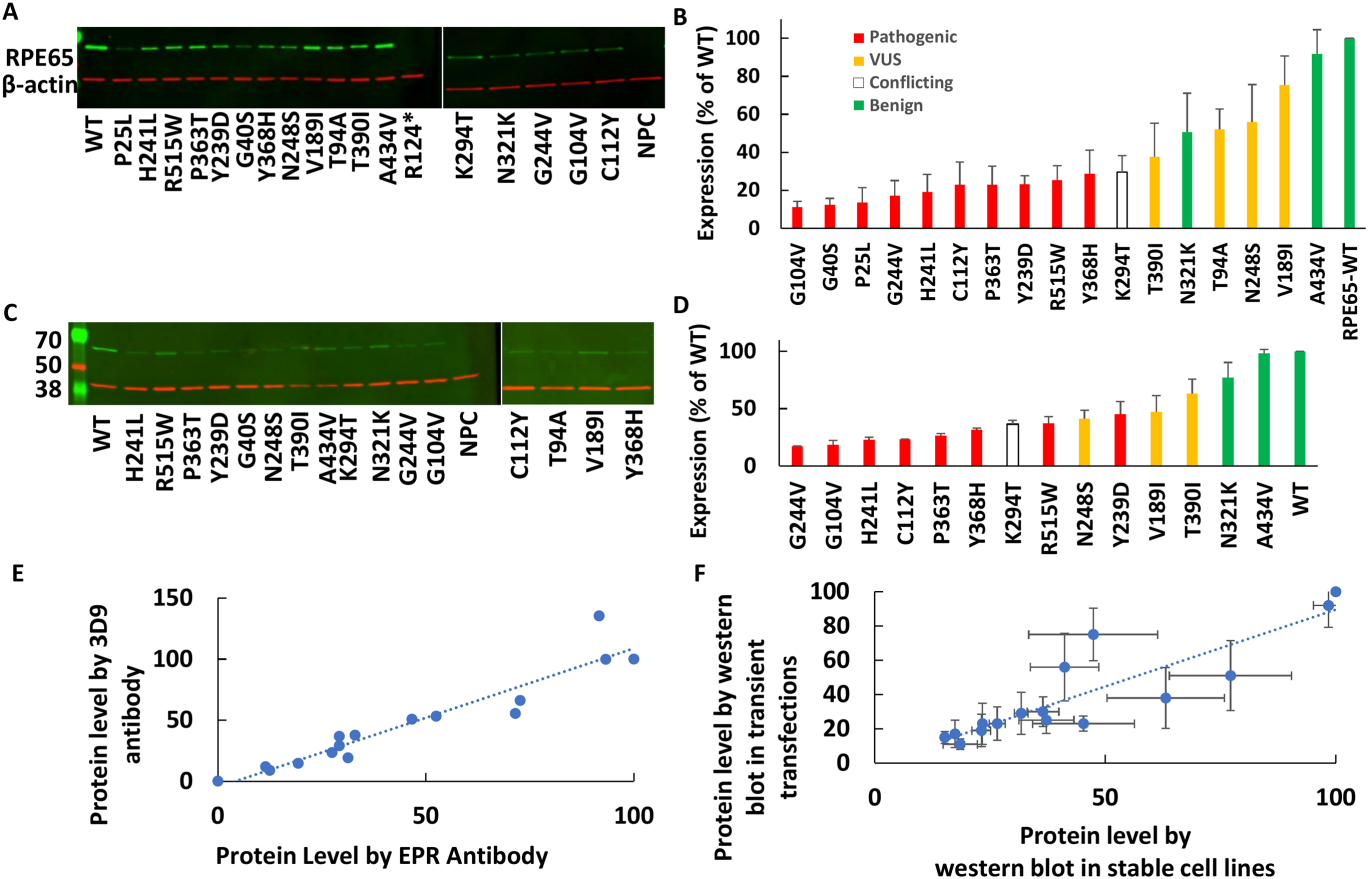
(**A–D**) Protein expression levels of different *RPE65* variants were assayed by western blotting: transient transfections (**A**) and stable cells lines (**C**), with corresponding densitometry results shown (**B**, **D**). In panels **B** and **D**, expression levels were normalized to β-actin levels and are expressed as a percentage of WT levels (*n* = 2 or 3), with known mutants shown in *red*, VUS in *yellow*, benign variants in *green*, and a variant with conflicting interpretations in *white*. (**E**) A high correlation was observed between the expression levels measured using two different *RPE65* antibodies (*r* = 0.95, *n* = 1). (**F**) A high correlation was observed between the average measurement of *RPE65* variants expressed by transient transfection and by stably expressing cell lines (*r* = 0.89, *n* = 2).

To calibrate the detection range and linearity of RPE65 and β-actin via western blotting, we prepared HEK293T cell lysates transiently transfected with WT *RPE65* and loaded 0.1 to 20 µg of whole cell lysates for immunoblotting (*n* = 2). Densitometry showed that the linear quantification range was narrow and required using relatively small amounts of protein (0.25–2 µg total lysate) and a small amount of antibody (1:5000 dilution of anti-*RPE65* EPR antibody), as seen in Figures 1A and 1B. There was no RPE65-specific band (∼61 kDa) observed in untransfected cell lysate (not shown; also see Fig. 2). To further delineate the proportional linear range, even smaller amounts of diluted protein lysate (0.05–4 µg) were used, which indicated that the proportional linear range for both RPE65 and β-actin (Santa Cruz Biotechnology, Dallas, TX, USA) were 0.1 to 2 µg (Figs. 1C, 1D). For stable cell lines that contained only a single copy of the *RPE65* transgene, there was an approximately fourfold lower expression in the stable lines compared to transient transfection, and protein lysate ranging from 1 to 20 µg was tested; 4 µg was at the higher end of the linear range (not shown). For further analysis of the different variants by western blot, 1 µg protein lysate for transient transfections and 4 µg protein lysate for stable cell lines were used per lane. Total protein staining (Revert 700 Total Protein Stain) was not accurately detectable by LICORbio Odyssey densitometry using the small protein amounts required for antibody detection within the linear range (not shown); therefore, β-actin was used as a loading control for normalization. For the single-copy stable cell lines only, the use of 4 µg protein lysate was slightly beyond the linear range of β-actin detection but was required for detection of RPE65. The use of 1 µg protein lysate from the transient transfections was within the linear detection range for both RPE65 EPR and β-actin. Similar results were obtained with the 3D9 RPE65 antibody, but for western blotting the linear range and detection limit were slightly lower (not shown). Altogether, these data indicated that the best combination of antibodies and conditions for the most accurate quantification of RPE65 protein levels by western blot was EPR (1:5000) and Santa Cruz anti-β-actin (1:5000; sc-47778) (see Materials and Methods). Therefore, these antibodies and concentrations were used for comparing *RPE65* variant expression levels using western blotting.

### Measurement of the Protein Levels of Different Variants by Western Blot in Transient Transfections and Stable Cell Lines

To analyze the effects of different variants of *RPE65* (benign positive controls, pathogenic negative controls, and VUS) on protein levels, western blotting was used to quantify protein levels in lysates from transient transfections (Figs. 2A, 2B) and stable cell lines (Figs. 2C, 2D). Using either transient transfection or stable cell lines, all of the pathogenic variants showed <50% of the WT protein level. Figure 2E shows a high correlation between staining of different variants with two different anti-RPE65 antibodies (*r* = 0.95), providing evidence for the specificity of the detected antigens. Also, the results showed a high correlation between measured protein levels in transiently transfected cells and in stable cell lines (*r* = 0.88) (Fig. 2F).

### RPE65 Protein Levels of Different Variants Using Flow Cytometry

**Figure 3.**
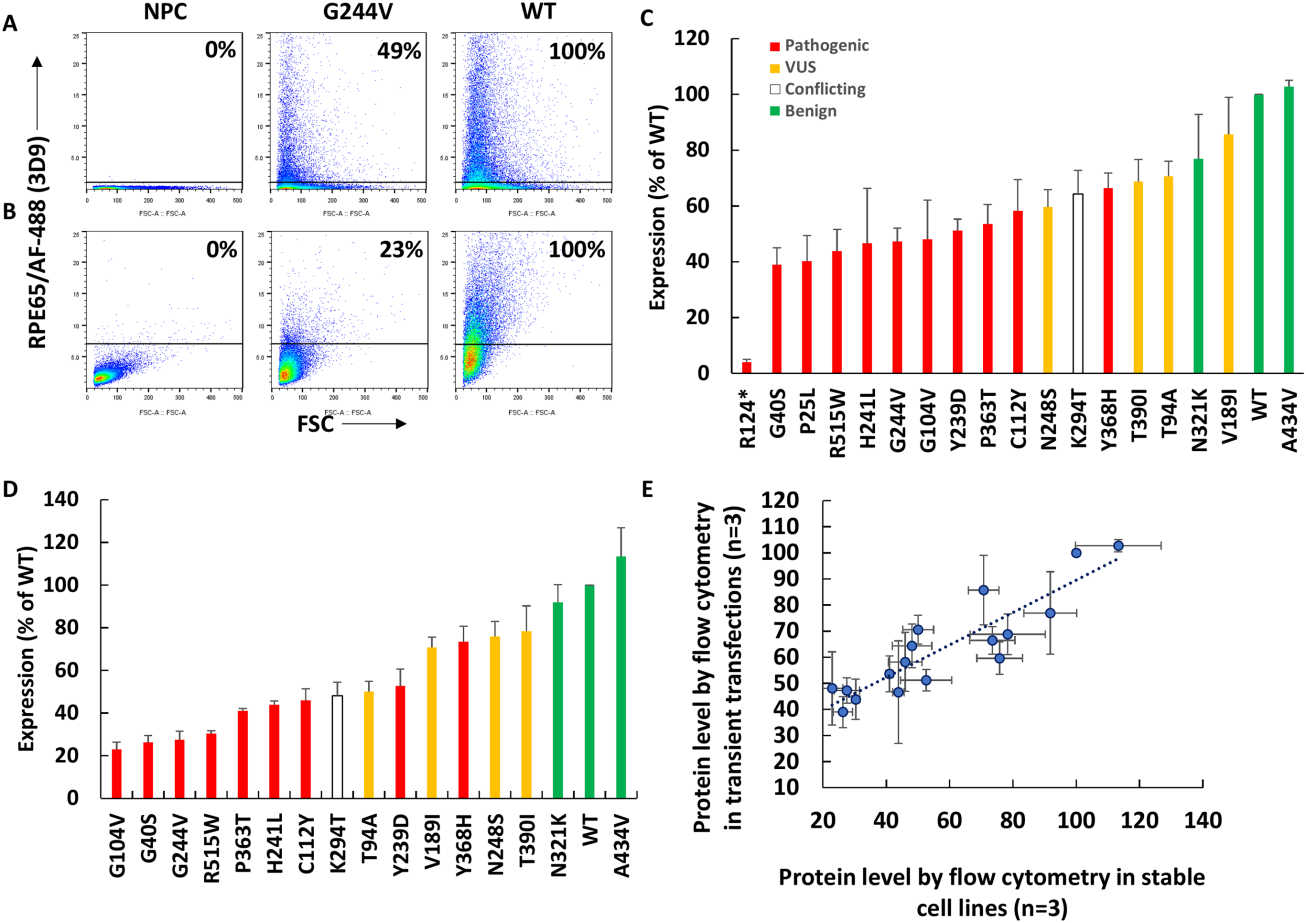
(**A**–**D**) Measurement by flow cytometry of protein levels of different variants in transient transfections (**A**, **C**) and stable cell lines (**B**, **D**). Shown are flow cytometry dot plots of untransfected cells (NPC), G244V, and WT *RPE65* in transiently transfected cells (**A**) and stable cell lines (**B**). The transiently transfected cells expressed higher antigen levels than the stable cell lines with single integrations. Quantification of protein levels of different variants (*red*, pathogenic; *yellow*, VUS; *green*, benign; *white*, conflicting) in transiently transfected cells (*n* = 3) (**C**) and stable cell lines (**D**) (*n* = 3). (**E**) High correlation can be seen in the measurement of protein levels by flow cytometry between transiently transfected cells and stable cell lines (*r* = 0.89, *n* = 3).

Flow cytometry was optimized to measure the protein levels of different *RPE65* variants using transient transfections and stable cell lines. Although the EPR antibody was best for western blotting, for flow cytometry antibody 3D9 showed a wider dynamic range than EPR for measuring the protein level across different variants (not shown). 3D9 was used for further staining for flow cytometry (Fig. 3). Flow cytometry results using transiently transfected cells and stable cell lines were highly correlated (*r* = 0.89, *n* = 3) (Fig. 3E).

### Correlations Between Assays

**Figure 4.**
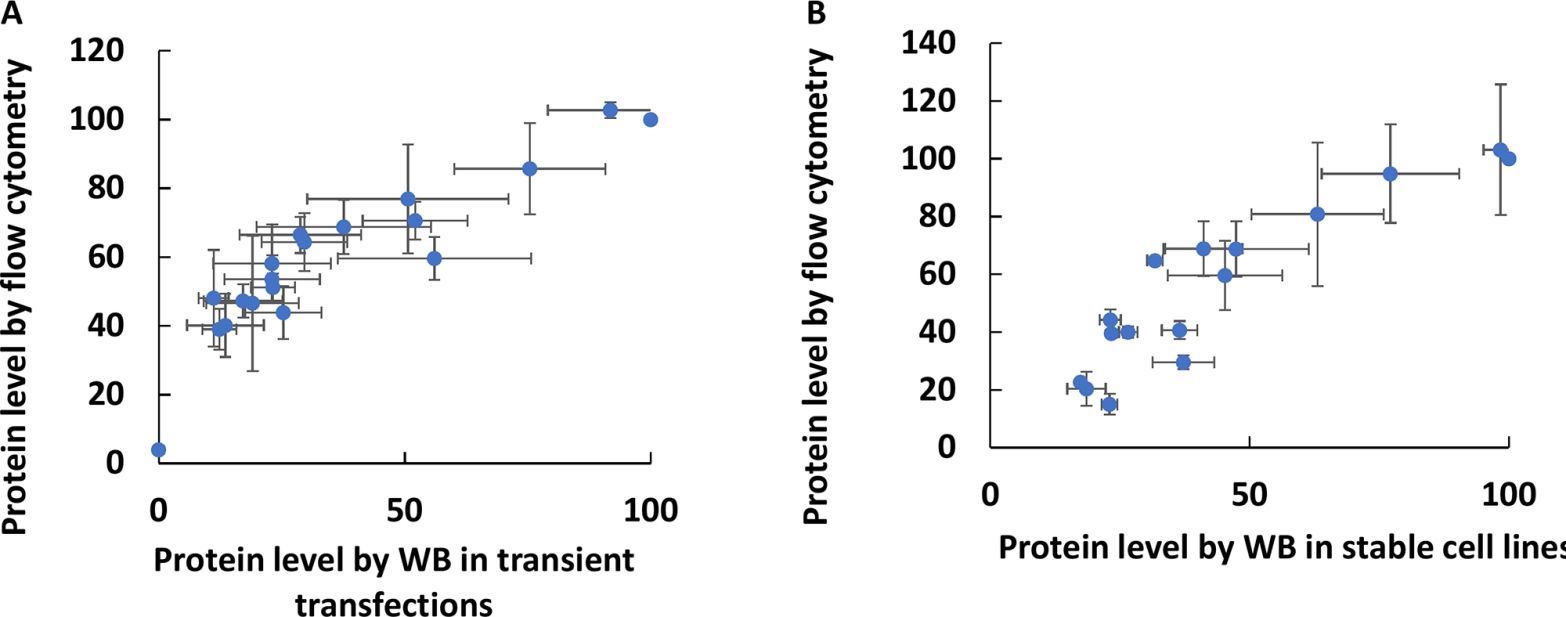
Optimized measurement of *RPE65* variant protein expression levels using western blotting with EPR antibody compared to flow cytometry with 3D9 antibody. (**A**, **B**) A high correlation was obtained in both transiently transfected cells (**A**) (*r* = 0.91, *n* = 3) and stable cell lines (**B**) (*r* = 0.91, *n* = 2).

**Figure 5.**
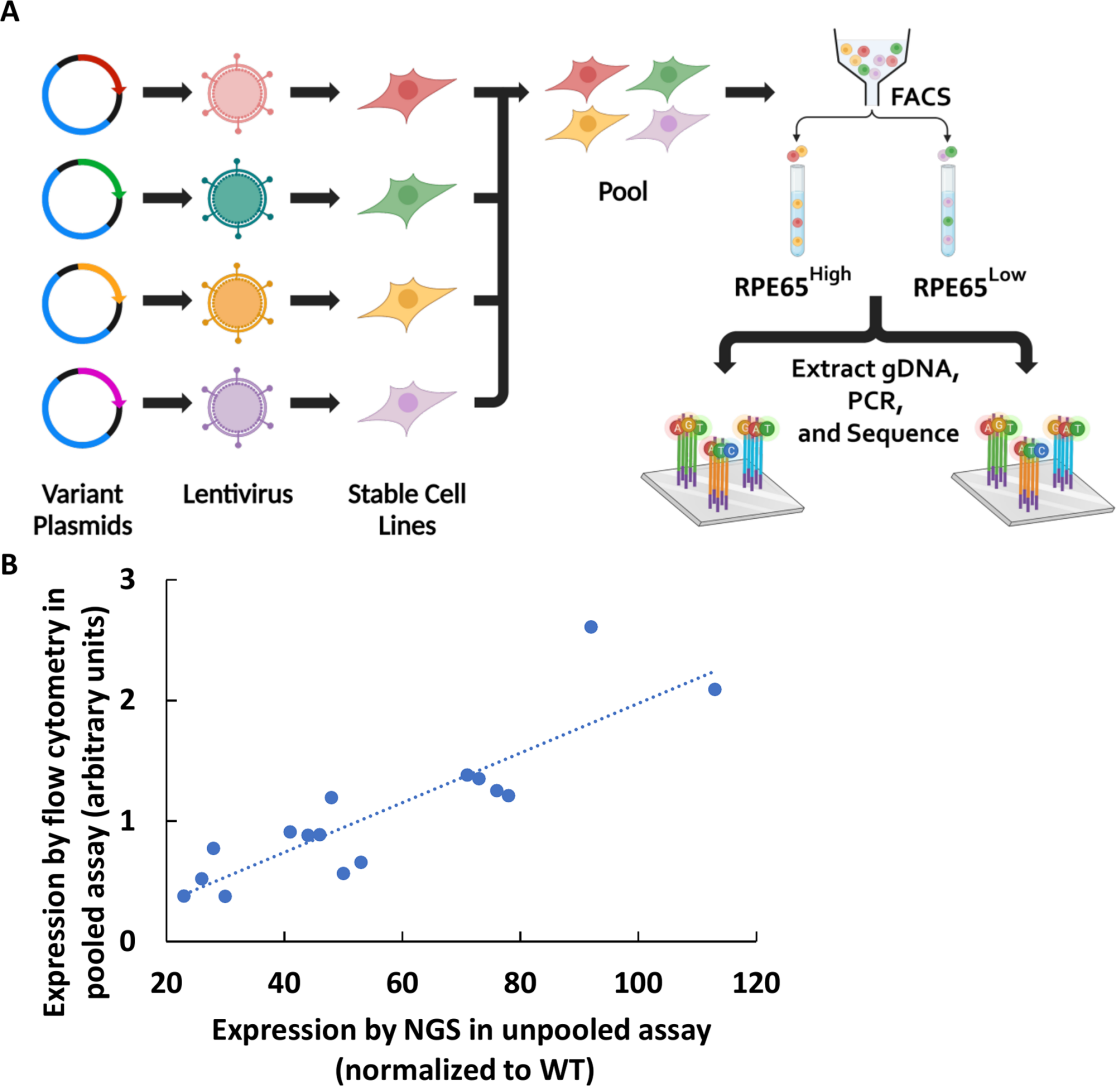
(**A**) Workflow of pooled library assay. (**B**) Comparing the measurement of expression levels in the pooled library by NGS and flow cytometry (unpooled). There was a high correlation between the pooled assay and unpooled flow cytometry assay (*r* = 0.87, *n* = 3).

Using western blotting for quantifying protein levels in cells is not easily scalable for measuring large numbers of different variant protein expression levels. Therefore, we optimized a flow cytometry assay to measure different variants, and this method showed high correlation to values measured by western blotting using transiently transfected cells (*r* = 0.91) (Fig. 4A) and stable cell lines (*r* = 0.91) (Fig. 4B). Next, a pooled assay was developed for a higher throughput flow cytometry assay based on pooled *RPE65* stable cell lines (Fig. 5A). A strong correlation was observed in the *RPE65* variant expression levels between the unpooled assay and the higher throughput pooled assay (*r* = 0.87) (Fig. 5B).

**Figure 6.**
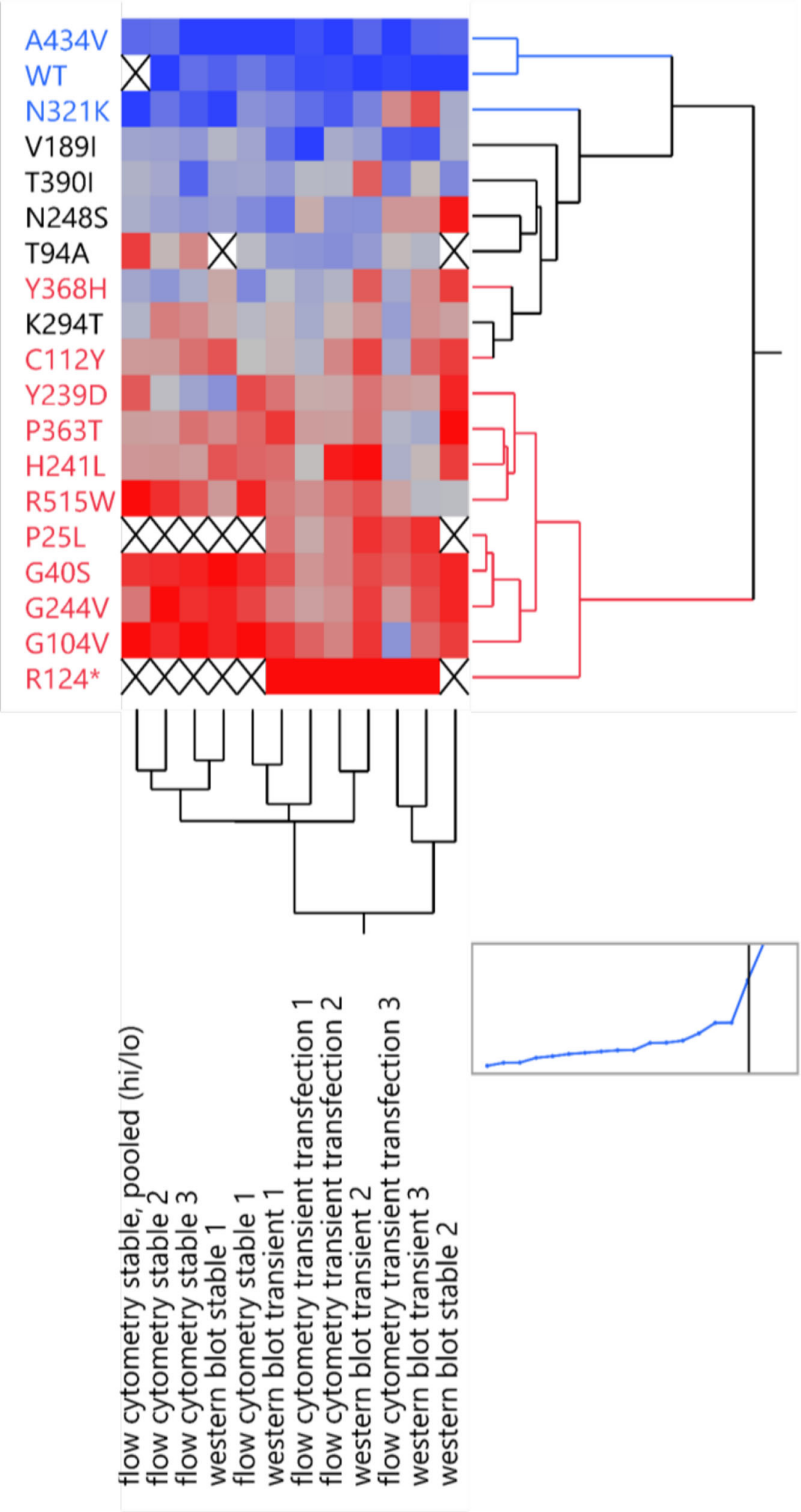
A heatmap comparing assay results across different assays (*columns*) and different *RPE65* variants (*rows*). A hierarchical clustering algorithm was used to group together similar data and produce row and column dendrograms. *Blue* denotes high expression, and *red* denotes low expression. An X indicates missing data.

In summary, the five assays tested above showed good agreement, as shown graphically in Figure 6, validating and giving support to the specificity and dynamic ranges of the assays tested above. The rare outlier points are also discussed below.

### Additional Variants Tested

**Figure 7.**
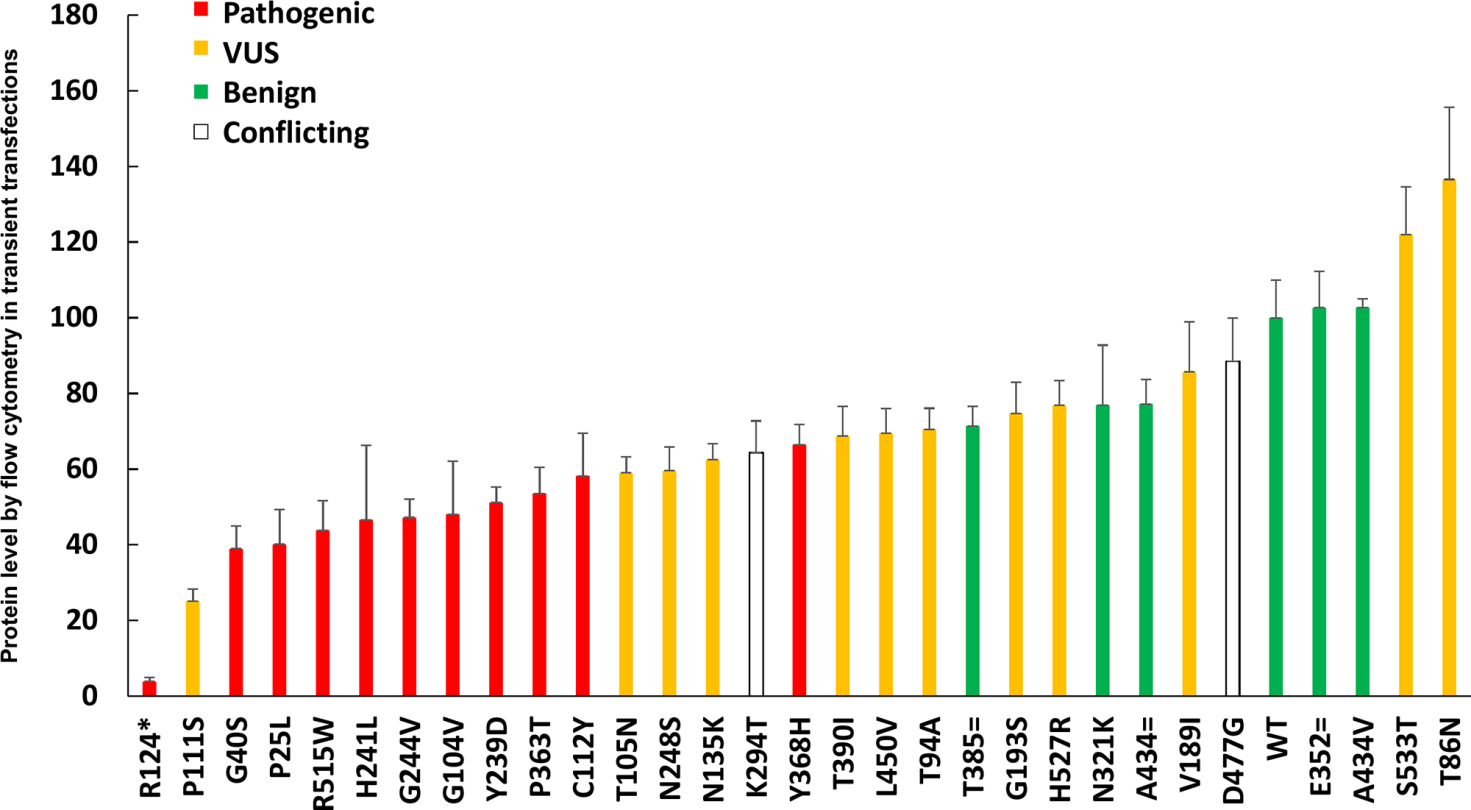
Protein levels of different *RPE65* variants measured by flow cytometry after transient transfection (*n* = 3). *Red* indicates pathogenic; *yellow*, VUS; and *green*, benign.

Next, based on the validation of the assays shown above, additional *RPE65* variants (see Table 2 and Materials and Methods) were assayed for protein expression levels using transient transfection, staining with the 3D9 antibody, and the unpooled flow cytometry readout. Figure 7 shows the expression levels of these additional variants. There was substantial but not complete separation between the values obtained by the positive controls (green) and the negative controls (red), as discussed below. P111S, initially a VUS, was identified as having pathogenic expression levels. Three positive control synonymous variants were hypothesized to have the same expression as the WT protein, but two, T385= and A434=, had slightly decreased levels (71% ± 5.23% and 77% ± 6.5%, respectively), in the context of their expression from a cDNA without introns.

### Enzymatic Assay for RPE65

**Figure 8.**
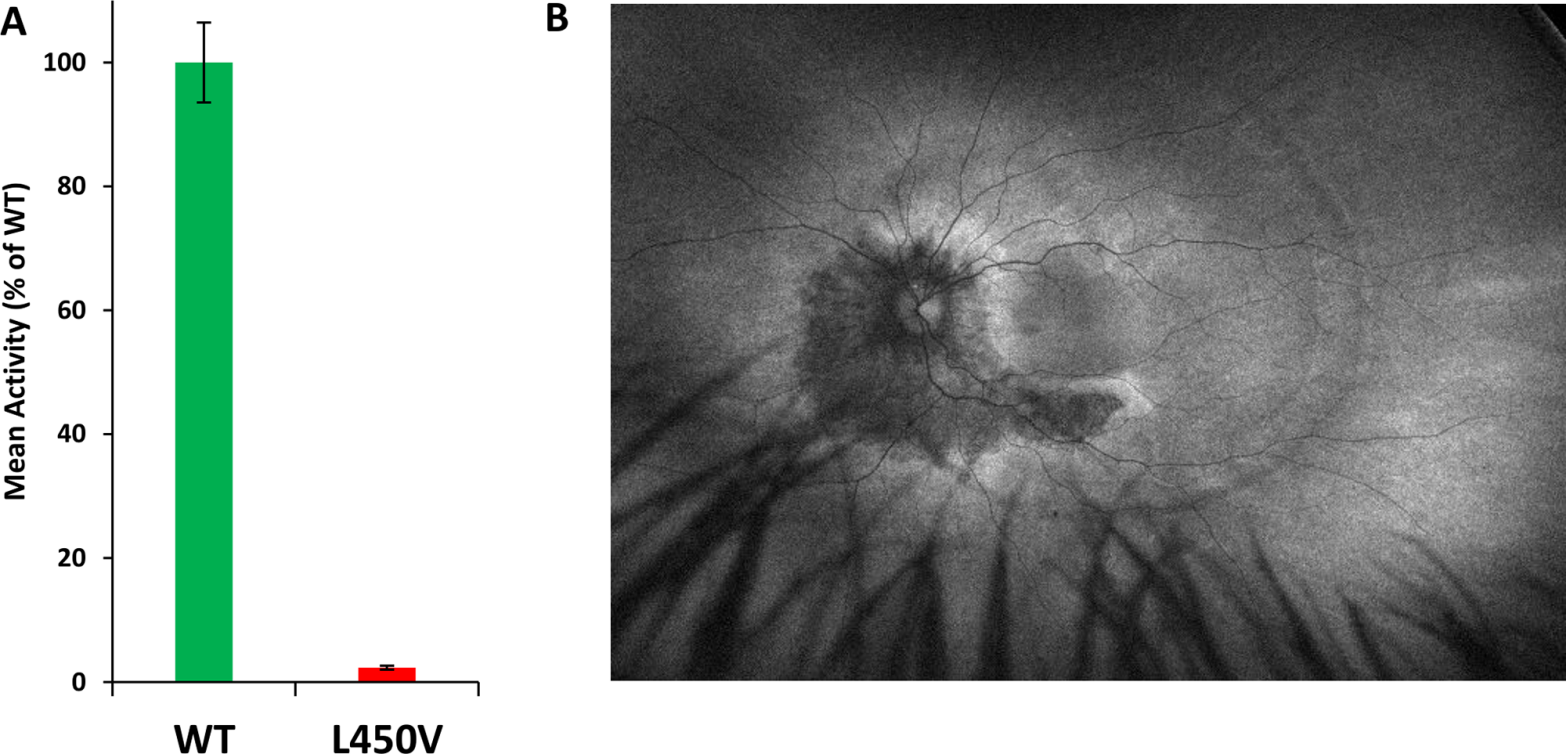
(**A**) Enzymatic activity assay results for RPE65 L450V. (**B**) This variant was seen in a patient with an atypical, mild phenotype, as revealed by autofluorescence imaging.

The fundus autofluorescence imaging of a patient with a compound heterozygous mutation L450V/R91W had an unusually mild phenotype with a slight increase in autofluorescence surrounding the optic nerve and subtle alterations in the retinal pigment epithelium (Fig. 8B). After normalization to WT levels, this variant was observed to have very low remaining enzymatic activity (2.3%, *n* = 3) (Fig. 8A).

## Discussion

With the approval of *RPE65* gene therapy and the increased number of IRD patients undergoing DNA sequencing, there is an increasing need for higher throughput pathogenicity assays for *RPE65* variants. The development of a higher throughput assay is often based on a simple but robust technique, and this study evaluated a straightforward protein detection assay to identify *RPE65* variants with pathogenic protein expression levels.

### Comparing the Protein Levels Measured in This Study With Those From Other Studies

By one estimate, 80% of missense mutations are misfolding, 10% are active site mutations, and 10% have another mechanism.^43^ Of course, these proportions and categories can vary by gene and remain to be fully defined for *RPE65*. Based on current understanding, the different mechanisms that can be involved in pathogenicity of different *RPE65* variants include misfolding, loss of catalytic activity, toxic gain of function, mislocalization, or aggregation of the mutated protein.

Regarding variants that are thought to be at or near the active site, we analyzed the expression level of three active site variants: H241L, H527R, and Y239D. The mechanism of pathogenicity in the active site is different than elsewhere,^31^ but interestingly our study showed that the protein levels of H241L and Y239D are also low. In fact, every pathogenic variant tested by western blot showed an expression level of 29% of WT or lower. The missense H527R variant was originally reported as a VUS in ClinVar and was observed in individuals with clinical features of *RPE65*-related conditions, but it has since been reclassified as pathogenic.^44^ *RPE65* with H527R mutation did not show any catalytic activity.^31^ This study showed a slightly decreased expression level (77%) (Fig. 7). Another variant close to active site cavity, Y239D, showed a pathogenic expression level by western blot and flow cytometry (Table 1). However, most disease-associated missense mutations in *RPE65* are non-active site mutations.^45^

Regarding the dominant variant D477G, this variant showed WT expression levels in HEK293T cells, consistent with a past study showing normal expression, localization, and catalytic activity in NIH3T3 cells.^46^ More recent studies have shown a decrease of expression levels in a knock-in mouse model^47^ and retinal degeneration only when exposed to environmental light.^48^

Regarding variants that are thought to cause misfolding and rapid degeneration of misfolded protein, G40S, R515W, G104V, and G244V showed significantly decreased protein levels compared to WT (≤29% of WT by western blot).^27^^,^^28^^,^^30^^,^^49^^,^^50^ Also, mouse models of the P25L and R91W mutations showed decreased protein levels of the mutants.^51^^,^^52^ This decrease in protein level is likely biologically relevant; the catalytic rate of RPE65 is so low that high expression of the protein plays the compensatory role for its weak enzymatic activity.^21^^,^^23^ The high stability of RPE65 (half-life >10 hours) and low degradation rate lead to high abundance in the retinal pigment epithelium.^53^

Regarding variants that may be benign, K294T was initially shown to have a severely decreased activity level.^23^ However, as seen in dbSNP (rs61752901; accessed 12/25) a high allele fraction in Latino populations of 1.1% to 3.1% indicates that it is benign. The K294T variant was reported in the heterozygous state in LCA patients.^30^ Unfortunately, both a later study^30^ and this study found borderline expression or activity levels. Although we believe that this variant is benign, the borderline levels do not allow that to be demonstrated definitively on a biochemical level, and we cannot rule out that it is hypomorphic. The other two benign variants in this study, A434V and N321K, showed normal expression.

Regarding variants that are in the amphipathic α-helix membrane targeting motif (amino acid residues 107–125), this study evaluated C112Y and P111S, which both showed pathogenic expression levels, with P111S showing the lowest expression of any missense variant tested in this study (Fig. 7). Residue C112 (studied as C112A by Uppal et al.^54^^,^^55^) plays an important role in palmitoylation and localization of the protein into the membrane.

### Interpreting the Meaning of RPE65 Expression Levels

The usual mechanism causing decreased protein levels of the pathogenic *RPE65* variants is proteasomal degradation of the misfolded protein.^31^ In this study and others, the relative tendency of a particular protein sequence to misfold should be proportional in heterologous cells compared to the disease target cell, although this was not formally tested in this study. The protein detection assay in this study was successfully scaled up to a pooled assay, which in future studies can be applied efficiently to a much larger number of *RPE65* variants. However, although the absence of protein expression is good evidence of a pathogenic mutation (and can provide immediately useful information in that case), a WT-like expression level does not guarantee that a variant is benign. Mutants with WT-like expression levels could be active site mutants or mislocalization mutants. Our laboratory has been exploring the development of a higher throughput RPE65 activity assay, intended to detect “active site” or other mutations that would affect enzyme activity but not protein levels. Despite our trying to include such mutations in the panel tested, Table 1 and Figure 6 show how the protein detection assay mirrors known activity levels surprisingly well. As the number of variants tested increases, however, it would be expected to find a small fraction of “false negative” results using a protein detection assay alone.

Table 1 summarizes the protein level of different variants in previous studies compared to the current study. Both Table 1 and Figure 6 show a broad agreement between both the literature values and the assays used in this study and among the different antibodies and techniques used. This lends higher confidence to the specificity and accuracy of the assays. The most robust and sensitive assay was western blotting using transient transfection, and the low expression levels of the stable cell lines were limited by the single-copy transgene and the presumed sensitivity of the antibody. In the pooled flow cytometry assay, the quantitative nature of counting the cells in the high versus low sorting gates, all in the same tube, is likely to increase the internal consistency of the assay compared to unpooled assays performed on separately stained and analyzed samples. Thanks to these advantages, the pooled assay gave good results (Fig. 6); however, the preferred antibody for flow cytometry, 3D9, had a moderate brightness/staining index. The dynamic range of the assay could be improved by developing a brighter antibody or, less ideally, using an epitope tag.

The assays in this study generally had broad agreement, but one specific variant (T94A) showed some disagreement between assays (Fig. 6). Specifically, T94A showed borderline levels in all unpooled assays but a low level in the pooled flow cytometry assay. Of note, the T94A stable cell line did not grow well, indicating a possible toxicity of transgene expression. Selection of unusual clones during the stable cell derivation could produce artifactual results, although this does not apply to transient transfections.

Regarding the determination of an expression level that should be considered pathogenic, the pathogenic mutants showed ≤30% of protein levels compared to WT by western blot of transiently transfected cells (Table 1), consistent with western blot results from other studies.^30^^,^^53^ Although the flow cytometry and western blot results showed a very high correlation (Fig. 4), the western blot values are likely more accurate on an absolute scale when comparing to external studies. By flow cytometry of transiently transfected cells, the dynamic range of the assay was slightly compressed, and less than 60% of WT expression was always pathogenic (Table 1; Fig. 7), with 60% to 70% in an intermediate zone and >70% always benign. In summary, pathogenic could be considered <30% of WT by western blot and <60% of WT by flow cytometry after transient transfection when using the protocols of this study. Although these simple numerical cutoffs are easy to understand and apply, as is often the case many such assays have an indeterminate region or “gray area.”^56^^,^^57^ Ideally, the probability of pathogenicity would be expressed as a quantitative (Bayesian) probability,^58^ but for *RPE65* this will require data across a larger number of variants. Adding data from an RPE65 activity assay would be beneficial, as well.

### Reclassifying Variants of Unknown Significance

Using the above criteria, P111S is solidly in the pathogenic range of expression levels. T105N, N248S, and N135K have borderline expression levels that may be pathogenic. V189I, S533T, and T86N show WT expression levels, but, without an activity assay, no strong conclusions can be made.

## Conclusions

All pathogenic *RPE65* variants tested show a low protein level, and validated protein expression assays can be used to reclassify the pathogenicity of VUS.^57^ Activity and localization assays would additionally identify the rare variants that have normal expression levels but lack activity. Future work may include using the pooled assay to assay hundreds or thousands of variants. This generation of functional data will aid in the diagnosis and treatment of patients with *RPE65*-associated retinal degeneration. Furthermore, developing formal rules for the use of such functional data for classifying the pathogenicity of variants is ongoing in the LCA/eoRD Variant Curation Expert Panel (VCEP) sponsored by the National Institute of General Medical Sciences, National Institutes of Health.

## Supplementary Material

Supplement 1
