# Supplementary material for: Higher Throughput Assays for Understanding the Pathogenicity of Variants of Unknown Significance in the RPE65 Gene
Source: Invest Ophthalmol Vis Sci. 2025 Oct 6;66(13):10. doi: 10.1167/iovs.66.13.10 (PMC12514977; doi:10.1167/iovs.66.13.10)
Supplement: Supplement 1 [file iovs-66-13-10_s001.pdf]

Supplemental Methods

Generation of stable cell line for *RPE65* variants: For lentiviral production, HEK293FT (Thermo Fisher, Cat no. R70007) cells were seeded at a density of $0.4x10^6$ cells/well of 6-well plate. Cloned pMT_025 *RPE65* lentiviral expression plasmid was co-transfected with pMD2.G (Addgene no. 12259) and psPAX2 (Addgene no. 12260) using Lipofectamine LTX transfection reagent (Thermo Fisher, Cat no. 1533803) following the manufacturer's instructions. Fresh media was supplemented after 24 hours and viral supernatant was harvested at 48, 72 and 96 hours, pooled, filtered and concentrated by using Lenti-X-Concentrator (Takara, Cat no.631232) according to the manufacturer's instructions. Concentrated aliquots were frozen in −80 °C until use. Titers were determined by transduction in HEK293T cells followed by Crystal Violet staining. For the generation of stable lines, $1x10^5$ HEK-293T cells were plated in a well of 6-well plate and transduced at an MOI of 0.3 by spinfection (2000g for 20 minutes) in the presence of Polybrene (8 μg/mL, Sigma, Cat no.107689). Media was replaced the following day. On day 3, puromycin (600 ng/mL) was added, selected for five days, and then expanded to establish stable lines. To confirm the integration, cells were lysed using Quick Extract Buffer (Lucigen, Cat no. QE09050), and PCR was performed using Q5 Master Mix (New England Biolabs, Cat no. M0492S) using primers XY304 (ATTCTCCTTGGAATTTGCCCTTT) and XY305 (CATAGCGTAAAAGGAGCAACA).

Quantification of *RPE65* levels using Western blots: Cell lysates were prepared for quantitating RPE65 protein levels 48 hours post-transfections. For stable cells lines, lysates were prepared at 80% confluency. Briefly, the cells were washed with PBS, trypsinized and washed again with cold PBS, and lysed using cold 1X RIPA buffer (Abcam, Cat no. ab156034) supplemented with protease inhibitor cocktails (Roche, Cat no. 11697498001). Lysates were

agitated at 4°C for 30 minutes and by briefly vortexing for 15 seconds, periodically every 10 minutes. Lysates were centrifuged at 18000×g for 30 minutes at 4°C and the supernatant was collected and stored at -80°C until use. Two variants (G40S, T94A) showed reduced growth rates in culture and, therefore, were excluded from the analysis.

Protein concentration was determined using Pierce BCA protein assay kit (Thermo Fisher Scientific, Cat no. 23225) according to the manufacturer's protocol. To assure correct quantitative immunoblots of *RPE65* different variants, different concentrations of whole cell lysate (0.1-20 µg) from wildtype *RPE65*-expressing cells were loaded onto the SDS-PAGE (4-20% tris-glycine gel, Invitrogen Cat no XP04200BOX) to find the linear range where the total protein loaded was linearly related to fluorescent band intensity. For electrophoresis loading, each sample was mixed with 4X LDS sample loading buffer (Invitrogen, Cat no. NP0007), denatured by heating at 70°C for 10 min, spun for 1 min at 18000×g and the supernatants were loaded onto the SDS-PAGE gel. Gel electrophoresis was performed at 150V for ~90 minutes. Then proteins were transferred from the gels onto polyvinylidene difluoride (PVDF) blotting membranes using the iBlot™ 2 system (Invitrogen, Carlsbad, CA) for 20V for 1 minute, 23V for 4 minutes and 25V for 2 minutes. Total protein staining was performed using Revert 700 following manufacturer's protocol (LICOR, Cat no.926-11011). For staining with *RPE65* and ß-actin antibodies, the membranes were blocked for 1 hour using Intercept® blocking buffer (LICOR, Cat no.927-60001), then incubated with 1:2500 or 1:5000 dilution of *RPE65* primary antibody and 1:5000 dilution of ß-actin primary antibody overnight at 4°C. The primary antibodies for *RPE65* were rabbit monoclonal [EPR7024(N)] anti-*RPE65* C-terminal (Abcam, ab175936), herein referred to as "EPR", and mouse monoclonal anti-*RPE65* (401.8B11.3D9) , (Novus Biologicals, Cat no. NB100-355), herein referred to as "3D9". For ß-actin, mouse

monoclonal IgG1 (Santa Cruz biotechnology, Cat no. sc-47778) and rabbit polyclonal (Abcam, Cat no. ab8227) were used. The following day, the membranes were washed 3 times with TBST (Tris buffered saline with 0.1% Tween-20) for 5 minutes and incubated with a 1:10,000 dilution of IRDye® 800CW and 700CW secondary antibodies for 1 hour followed by three 5 min washes in TBST. The secondary antibodies were IRDye 680RD Goat anti-Mouse (LICOR, Cat no. 926-68070) and IRDye 800CW Goat anti-Rabbit (Licor , Cat no. 926-32211). The membranes were then imaged on the 800-nm and 700-nm wavelength channel using the Odyssey CLx infrared imaging system (LICOR, Lincoln, Nebraska). The density of Western blot bands was measured using Odyssey software and normalized to ß-actin internal protein levels. After determining the proportional linear range for detection of ß-actin and *RPE65*, Western blots of lysates from *RPE65* transient transfection and stable cell lines were performed using 1µg of total protein for transient transfections and 4µg of total protein for stable cell lines, as described above. All data were then normalized to the expression level seen in the wild-type (WT) *RPE65* sample.

Quantification of RPE65 expression levels by flow cytometry

Initial experiments used the PETLET RPE65 antibody (not shown; from the Redmond laboratory), but the following experiments were conducted with commercially available antibodies that are more broadly available. Cells were collected for flow cytometry analysis 48 hours post transfection. For stable cells lines, cells were collected at 80% confluency. After washing with PBS, cells were collected by trypsinization, centrifuged at 1200 g for 4 minutes, and washed with PBS. The cells were resuspended and fixed in 4% paraformaldehyde (PFA, Electron Microscopy Sciences, Cat no. 15714) in PBS for 20 min and washed with PBS and kept at 4 °C until staining. For staining of *RPE65*, cells were permeabilized with 0.02% saponin (Sigma, Cat no. 47036) in PBS for 12 minutes, followed by blocking in 3% BSA in PBS. After

blocking, cells were incubated with primary antibodies described above at a final concentration of 1:1500 in blocking buffer for 1 hour. After washing with PBS, Alexa fluor-488 secondary antibodies goat anti-mouse IgG1 (Invitrogen, Cat no. A21121) and goat anti-rabbit IgG (Invitrogen, Cat no. A27034) were used at a final concentration of 1:500 in blocking buffer for 1 hour. After a PBS wash, cells were resuspended in appropriate volume of PBS and analyzed on a Miltenyi BioTec MACSQuant flow cytometer. Flow cytometry results were analyzed with FlowJo Software v10. The resulting data were averaged across multiple independent transfections (N= 3-5).

Antibody List:

Flow cytometry:

Anti-RPE65 mouse monoclonal, (Novus Biologicals, Cat no. NB100-355) "3D9", 1:1500

Alexa fluor-488 secondary goat anti-mouse IgG1 (Invitrogen, Cat no. A21121), 1:500

Alexa fluor-488 secondary Goat anti-rabbit IgG (Invitrogen, Cat no. A27034), 1:500

Western blotting:

Anti-RPE65 rabbit monoclonal EPR7024(N) anti-*RPE65* C-terminal (Abcam, ab175936) "EPR", 1:5000

Anti ß-actin mouse monoclonal IgG1 (Santa Cruz biotechnology, Cat no. sc-47778), 1:5000

Anti ß-actin rabbit polyclonal (Abcam, Cat no. ab8227)- only initially used with 3D9 primary; not in final assay

IRDye 680RD Goat anti-Mouse (LICOR, Cat no. 926-68070), 1:10,000

IRDye 800CW Goat anti-Rabbit (Licor , Cat no. 926-32211), 1:10,000

No.	Transcript consequence *RPE65* (NM_000329.3)	Protein consequence
1	c.1580A>G	p.His527Arg
2	c.577G>A	p.Gly193Ser
3	c.1348C>G	p.Leu450Val
4	c.1430A>G	p.Asp477Gly
5	c.1597T>A	p.Ser533Thr
6	c.314C>A	p.Thr105Asn
7	c.405T>A	p.Asn135Lys
8	c.257C>A	p.Thr86Asn
9	c.1155G>A	p.Thr385=
10	c.331C>T	p.Pro111Ser
11	c.722A>T	p.His241Leu
12	c.1543C>T	p.Arg515Trp
13	c.1087C>A	p.Pro363Thr
14	c.715T>G	p.Tyr239Asp
15	c.118G>A	p.Gly40Ser
16	c.1102T>C	p.Tyr368His
17	c.743A>G	p.Asn248Ser
18	c.565G>A	p.Val189Ile
19	c.280A>G	p.Thr94Ala
20	c.1169C>T	p.Thr390Ile
21	c.1301C>T	p.Ala434Val
22	c.881A>C	p.Lys294Thr
23	c.963T>G	p.Asn321Lys
24	c.731G>T	p.Gly244Val
25	c.311G>T	p.Gly104Val
26	c.335G>A	p.Cys112Tyr
27	c.74C>T	p.Pro25Leu
28	c.370C>T	p.Arg124*
29	c.1302G>A	p.Ala434=
30	c.1056G>A	p.Glu352=

Supplementary Table 1. List of different *RPE65* variants tested in this study with HGVS nomenclature. The reference transcript is NM_000329.3.

No.	Variants	Pathogenicity	Literature expression %	Literature activity %	References
1	Thr101Ile	Pathogenic	~100, ~15	1.27, 2.4	30,31
2	Leu408Pro	Pathogenic	~80, ~20	5.86, 5	30,31
3	Tyr79His	Pathogenic	3.31	2.5	25
4	Glu95Gln	Pathogenic (mild)	28.07	6.1	25
5	Leu22Pro	Pathogenic	32.4, ~20	13.5, 3.9	25,31
6	Arg44Gln	Pathogenic	~75, ~25	<2, 1.34	23,30,31
7	Arg91Gln	Pathogenic	~110	1.33	30
8	His68Tyr	Pathogenic		<2	23
9	Ala132Thr	Pathogenic		50	23
10	His182Tyr	Pathogenic		10	23
11	Glu417Gln	Pathogenic		<2	23
12	Gly528Val	Pathogenic		<2	23
13	Cys330Tyr	Pathogenic	0	<2, 1.6, 1.6	31,45,59
14	Ala434Val	Benign		55	23
15	Glu417Gln	Pathogenic	~75	1.1	26,31
16	His313Arg	Pathogenic		Not Detectable	31
17	Tyr318Asn	Pathogenic	~20, ~20	5.12, 7.1	30,31
18	Arg91Trp	Pathogenic	~10	5.08	30

Supplementary Table 2. Literature review of expression levels and catalytic activities of additional *RPE65* variants.
